# Serum IgE, IL-4 and IL-13 Concentrations as Th2-Associated Biomarkers in Canine Giardiasis

**DOI:** 10.3390/vetsci13100998

**Published:** 2026-09-23

**Authors:** Murat Kaan Durgut, Mahmut Ok, Süleyman Serhat Iyigun, Hazal Akkus, Gamze Sivrioglu

**Affiliations:** Department of Internal Medicine, Faculty of Veterinary Medicine, Selcuk University, Konya 42000, Turkey; mok@selcuk.edu.tr (M.O.); serhat.iyigun@selcuk.edu.tr (S.S.I.); hazalakkus0711@gmail.com (H.A.); gamzesvrl@gmail.com (G.S.)

**Keywords:** canine giardiasis, Th2 immune response, interleukin-4, interleukin-13, immunoglobulin E

## Abstract

*Giardia duodenalis* is a common intestinal protozoan parasite in dogs, but the systemic immune response associated with canine giardiasis remains incompletely characterized. This study aimed to evaluate serum concentrations of immunoglobulin E (IgE), interleukin-4 (IL-4), and interleukin-13 (IL-13) as Th2-associated immune markers in naturally infected dogs. Thirty client-owned dogs with confirmed giardiasis and 10 clinically healthy control dogs were included in the study. Serum IgE, IL-4, and IL-13 concentrations were measured using canine-specific enzyme-linked immunosorbent assays. Between-group comparisons were performed using two-sided Mann–Whitney U tests, with Holm adjustment for multiple comparisons, and effect estimates were calculated using the Hodges–Lehmann method. Spearman’s rank correlation was used to assess associations among biomarkers within each group. Serum IL-4 concentrations were significantly higher in dogs with giardiasis than in control dogs (median 38.12 [IQR 32.55–57.97] vs. 23.65 [18.86–27.90] pg/mL; Mann–Whitney U = 238.5, Holm-adjusted *p* < 0.001), with a Hodges–Lehmann estimated location shift of 16.31 pg/mL (95% CI: 9.80–27.47). Serum IgE concentrations were higher in dogs with giardiasis but did not remain statistically significant after Holm adjustment (1.32 [1.15–1.62] vs. 1.16 [1.02–1.33] µg/mL; Holm-adjusted *p* = 0.125), while IL-13 concentrations did not differ between groups (78.23 [53.05–103.42] vs. 79.05 [65.44–103.68] ng/L; Holm-adjusted *p* = 0.988). Within the giardiasis group, IgE, IL-4, and IL-13 concentrations were significantly and positively correlated with one another (ρ = 0.644–0.690, *p* < 0.001). In control dogs, only IL-4 and IL-13 were significantly correlated (ρ = 0.636, *p* = 0.048). These findings provide evidence consistent with altered Th2-associated immune activity in canine giardiasis, characterized by a significant elevation of serum IL-4 and coordinated variation among Th2-associated biomarkers. The non-significant findings for serum IgE and IL-13 may reflect a selective pattern of systemic Th2-associated biomarker expression, although limited statistical power prevents exclusion of smaller between-group differences. Further studies incorporating intestinal mucosal measurements and longitudinal assessment are warranted to clarify the relationship between systemic and local immune responses in canine giardiasis.

## Simple Summary

*Giardia duodenalis* is a common intestinal parasite in dogs and may cause diarrhea and other gastrointestinal disturbances, particularly in puppies. The clinical outcome of infection is closely associated with the host immune response. Although a Th2-associated immune response has been implicated in host defence against *Giardia* in humans and experimental models, limited information is available regarding this response in naturally infected dogs. In this study, serum IgE, IL-4, and IL-13 concentrations were compared between 30 dogs naturally infected with *G. duodenalis* and 10 healthy control dogs. Serum IL-4 concentrations were significantly higher in dogs with giardiasis than in healthy controls, and this difference remained significant after correction for multiple comparisons; IgE concentrations showed a similar upward trend that did not reach statistical significance after correction, and IL-13 concentrations did not differ between the groups. Positive correlations among IgE, IL-4, and IL-13 suggested coordinated variation among these Th2-associated biomarkers. These findings provide evidence consistent with altered Th2-associated immune activity in canine giardiasis, primarily reflected by significantly increased serum IL-4 concentrations, despite the absence of significant differences in serum IgE and IL-13 concentrations.

## 1. Introduction

*Giardia intestinalis* (*Giardia duodenalis*, *Giardia lamblia*) is a flagellated protozoan causing giardiasis, one of the most common parasitic infections in humans and mammals worldwide [1]. Infection is transmitted by the fecal–oral route, and sources of *G. duodenalis* infection include infected individuals, direct contact with animals, and contaminated water or food. *Giardia* is also one of the most common parasites found in dogs. The prevalence of *G. duodenalis* in dogs has been reported to range from 1.6% to 100% across different geographic regions [2]. *Giardia* infection is more common in young dogs and shelters. Clinical signs of acute giardiasis include malabsorption, diarrhea, abdominal pain, and weight loss. However, *Giardia* infection is typically asymptomatic and has a chronic course [1]. The development of clinical signs depends on many factors, primarily the host’s immunity. Severe cases of giardiasis are observed in young or immune-suppressed animals [3].

*G. duodenalis* has a direct life cycle. Transmission occurs fecal–orally, by direct contact, or through contact with contaminated materials [4]. In *Giardia* infections, villus atrophy, microvillus shortening, decreased disaccharidase activity, loss of epithelial barrier function, increased intestinal permeability, and mucosal damage are observed [5].

*Giardia* antigens may contain allergen-like molecules that increase the production of total and specific IgE [6]. The cross-linking of IgE on mast cells and eosinophils via FcεRI triggers their activation and subsequently initiates the release of mediators such as cytokines, histamine, and leukotrienes. These molecules may play aroles in clearing the parasite and even in the development of allergies [7]. It has been reported that this resulting immune response may serve as a protective mechanism against the disease and may be mediated by T cells and IgA antibodies [8,9]. Cytokines are small soluble proteins that modulate cell differentiation, activation, and proliferation. The type and amount of cytokine responses may be influenced not only by variations in the duration and severity of the infection, but also by the high antigenic variability of specific *Giardia* surface proteins [10,11].

IL-13 is a cytokine produced primarily in response to the Th2 response associated with intestinal inflammatory conditions and allergies. It plays a key role in stimulating mucus-related secretory activity of goblet cells in the intestinal mucosa, contributing to eosinophil activation and increased IgE production and secretion. It has been reported that IL-13 release is increased in giardiasis, resulting in triggered IgE production [12,13]. In contrast, Weatherhead et al. [14] reported that there was no difference in serum IL-13 concentrations between children with giardiasis and healthy controls. IL-4 is the primary cytokine produced during Th2 responses, and its levels increase during infections. Elevated IL-4 levels have also been observed in giardiasis [15,16,17]. It has been reported that cytokines show a two-phase response during the course of a giardiasis infection, with Th1 (IFN-γ, IL-1β, IL-6, TNF)- and Th2-mediated IL-4 release during the acute phase [18].

It has been demonstrated that IgE plays an important role in immunity against parasitic infections [7]. Total IgE levels have been increased in giardiasis [19], and it has been reported that *Giardia* proteins may be responsible for this increase because they induce an IgE response [17]. On the other hand, some studies conducted on children with giardiasis have reported no difference in total IgE concentrations compared to control groups [20,21]. However, these studies did identify a positive association between *Giardia* and skin allergies [20]. It has been suggested that the increase in the incidence of urticaria and food allergies may be a result of the immune response in giardiasis, and it has been reported that damage to the intestinal mucosa may lead to the absorption of foreign substances and the development of allergies [19]. It has been reported that patients with giardiasis who did not exhibit allergic symptoms showed no statistically significant difference in total IgE concentrations [20].

Although the immune response to *Giardia* infection has been studied in humans and experimental animal models, limited data is available on the cytokine response in naturally infected dogs. We hypothesized, and aimed to determine, whether dog naturally infected with *Giardia duodenalis* dogs show altered serum IgE, IL-4 and IL-13 concentrations consistent with a Th2-associated immune response.

## 2. Materials and Methods

The study was conducted between February 2026 and August 2026 in a total of 40 dogs at Selcuk University, Faculty of Veterinary Medicine, Small Animal Hospital. The study protocol was approved by the Institutional Ethics Committee of the Faculty of Veterinary Medicine, Selçuk University (approval no. 2026/33), and informed owner consent was obtained.

### 2.1. Animals

A total of 40 client-owned dogs were included in this study. The animals were divided into two groups: a giardiasis group (*n* = 30) and a healthy control group (*n* = 10). Eighteen of the dogs were female, and 22 were male. Eighteen dogs were mixed breeds, 7 were Kangal shepherd dogs, 5 were terriers, 4 were King Charles Cavaliers, 3 were Golden Retrievers, and 3 were German Shepherds. The average age of the dogs was 8.2 months.

#### 2.1.1. Dogs with Giardiasis

The giardiasis group consisted of dogs naturally infected with *Giardia duodenalis*, diagnosed by zinc sulfate fecal flotation based on the detection of *Giardia* cysts in three consecutive fecal samples collected from each dog. Dogs with concurrent parasitic infections identified by coproparasitological examination, including toxocariasis, isosporiasis, and other intestinal parasitoses, were excluded from the study. In addition, dogs that tested positive for canine parvovirus or canine coronavirus using a rapid diagnostic test (Asan Easy Test Corona Parvo^®^, Asan Pharmaceutical, Seoul, Republic of Korea) were not included. Animals that had received vaccination or antiparasitic treatment within 3 weeks prior to sampling were also excluded. Of the 30 dogs in this group, 16 were male and 14 were female, with ages ranging from 2 months to 4 years (mean 8.4 months). Breeds included 14 mixed-breed dogs, 5 Kangal shepherd dogs, 3 terriers, 4 King Charles Cavaliers, 1 Golden Retriever, and 3 German Shepherds.

#### 2.1.2. Healthy Control Dogs

The healthy group consisted of clinically healthy dogs aged between 2 months and 5 years. Inclusion criteria for the control group were the absence of *Giardia* cysts in three consecutive fecal examinations performed by zinc sulfate flotation, negative coprological findings for other intestinal parasites, and negative serological results for canine parvovirus and coronavirus infections. All control dogs were free of clinical signs suggestive of gastrointestinal disease at the time of examination. Of the 10 dogs in this group, 6 were male and 4 were female, with ages ranging from 2 months to 5 years (mean 7.6 months). Breeds included 4 mixed-breed dogs, 2 Kangal shepherd dogs, 2 terriers, and 2 Golden Retrievers.

### 2.2. Collection of Fecal Samples

Approximately 5 g of fecal material was obtained directly from each dog via rectal sampling and divided into two separate containers. One portion of each sample was immediately transported to the Laboratory of Parasitology, Faculty of Veterinary Medicine, Selçuk University, for coproparasitological evaluation, including native microscopic examination and flotation techniques. The second portion of the fecal sample was used for the detection of canine parvovirus and coronavirus antigens using a commercial rapid immunochromatographic test kit (Asan Easy Test Corona Parvo^®^, Asan Pharmaceutical, Seoul, Republic of Korea), according to the manufacturer’s instructions.

### 2.3. Collection of Blood Samples

Venous blood samples were aseptically collected from the cephalic vein of all dogs. Part of each sample was transferred into 8 mL serum collection tubes for serum preparation. Serum samples were separated by centrifugation at 2000× *g* for 5 min after clot formation and preserved at −80 °C until laboratory analyses were performed.

### 2.4. Biomarker Analyses

Serum IgE, IL-4 and IL-13 concentrations (Bioassay Technology Laboratory Co., Jiaxing, China) were measured with commercial canine-specific ELISA test kits according to the manufacturer’s instructions. Canine IgE commercial ELISA kit (Bioassay Technology Laboratory Co., Jiaxing, China, Cat. No.: E0208Ca), canine IL-4 commercial ELISA kit (Bioassay Technology Laboratory Co., Jiaxing, China, Cat. No: E0003Ca), and canine IL-13 commercial ELISA kit (Bioassay Technology Laboratory Co., Jiaxing, China, Cat. No.: E0351Ca) were used for ELISA analyses of biomarkers. The intra-assay coefficient of variation (CV), inter-assay CV, and minimum detectable concentrations (MDCs) for biomarkers were <8%, <10%, 0.026 µg/mL for IgE, <8%, <10%, 1.02 pg/mL for IL-4, and <8%, <10%, 3.18 ng/L for IL-13, respectively.

### 2.5. Statistical Analysis

Statistical analyses were performed using SPSS version 25 (IBM Corp., 2017, Armonk, NY, USA). The Shapiro–Wilk test was used to assess the normality of the data. Because the biomarker data showed departures from normality and the sample sizes were relatively small, nonparametric methods were used for between-group comparisons. Two-sided Mann–Whitney U tests were used to compare serum IgE, IL-4, and IL-13 concentrations between the giardiasis and control groups. The Hodges–Lehmann estimate of the location shift between groups, together with its 95% confidence interval, was calculated as an effect measure for each comparison. Data are expressed as median [interquartile range, IQR]. Values identified as possible outliers by the IQR rule were retained in the analysis, as there was no evidence that they resulted from data-entry error. Spearman’s rank-order correlation coefficient (ρ) was used to assess associations among serum IgE, IL-4, and IL-13 concentrations within the giardiasis and control groups separately. Correlations were not calculated using the pooled sample. Spearman’s correlation was selected because several variables, particularly IgE, showed departures from normality. These within-group correlation analyses were considered exploratory, and no multiplicity adjustment was applied.

For between-group comparisons, a two-sided *p*-value < 0.05 was considered statistically significant. Because three biomarkers were compared between groups, Holm’s sequential correction was applied to control for multiple comparisons; both unadjusted and Holm-adjusted *p*-values are reported to allow assessment of the robustness of the findings.

## 3. Results

### 3.1. Clinical Findings

Dogs diagnosed with giardiasis presented primarily with gastrointestinal disturbances of varying severity. The most common clinical sign was diarrhea, characterized by soft to watery feces frequently containing excessive mucus. According to owner reports, the duration of diarrhea ranged from several days to more than one week before presentation. All dogs in the giardiasis group were clinically symptomatic. Hematochezia was observed in 17/30 dogs (56.7%), and reduced appetite or intermittent anorexia in 11/30 dogs (36.7%); decreased activity levels were also noted among affected dogs.

### 3.2. Biomarker Findings

Biomarker findings of controls and dogs with giardiasis are presented in Table 1 and Table 2a,b. Serum IgE concentrations were higher in dogs with giardiasis than in control dogs (median 1.32 [IQR 1.15–1.62] vs. 1.16 [1.02–1.33] µg/mL, respectively), but this difference was not statistically significant after Holm correction (Mann–Whitney U = 189.5, *p* = 0.062, Holm-adjusted *p* = 0.125). Serum IL-4 concentrations were significantly higher in dogs with giardiasis than in control dogs (median 38.12 [IQR 32.55–57.97] vs. 23.65 [18.86–27.90] pg/mL, respectively; Mann–Whitney U = 238.5, *p* < 0.001, Holm-adjusted *p* < 0.001), with a Hodges–Lehmann estimated location shift of 16.31 (95% CI: 9.80–27.47). Serum IL-13 concentrations did not differ significantly between dogs with giardiasis and control dogs (median 78.23 [IQR 53.05–103.42] vs. 79.05 [65.44–103.68] ng/L, respectively; Mann–Whitney U = 149.5, *p* = 0.988, Holm-adjusted *p* = 0.988).

### 3.3. Correlation Analysis

Correlations among IgE, IL-4 and IL-13 were computed separately within each group (Table 2a,b). Within the giardiasis group (*n* = 30), all three biomarkers were significantly and positively intercorrelated: IgE with IL-4 (ρ = 0.644, *p* < 0.001), IgE with IL-13 (ρ = 0.690, *p* < 0.001), and IL-4 with IL-13 (ρ = 0.672, *p* < 0.001). Within the control group (*n* = 10), only IL-4 and IL-13 were significantly correlated (ρ = 0.636, *p* = 0.048); IgE was not significantly correlated with either IL-4 (ρ = 0.188, *p* = 0.603) or IL-13 (ρ = 0.030, *p* = 0.934) in control dogs, although the small control sample limits the power to detect such an association.

## 4. Discussion

In this study, we found that serum IL-4 concentrations in dogs naturally infected with *Giardia duodenalis* were significantly higher than those in the healthy control group (Mann–Whitney U = 238.5, Holm-adjusted *p* < 0.001), and this difference remained significant after correction for multiple comparisons. In contrast, no statistically significant difference was found between dogs with giardiasis and healthy dogs in terms of serum IL-13 and IgE concentrations (Holm-adjusted *p* ≥ 0.125). Furthermore, within the giardiasis group, significant positive correlations were found between serum IgE levels and IL-4 and IL-13 levels.

*Giardia duodenalis* is a non-invasive protozoan parasite that settles in the intestinal mucosa without invading the epithelial layer. Therefore, infection typically causes minimal tissue damage, and clinical presentation depends primarily on the nature of the host–parasite interaction rather than the parasite’s direct effects [22]. However, the presence or absence of symptoms and the severity of the disease are largely determined by the host’s immune response [3]. Both Th1 and Th2-type immune mechanisms have been reported to play roles in *Giardia* infections, with these two responses being activated to varying degrees during different stages of the infection [18]. Thus, evaluating cytokines and associated molecules (e.g., IL-4, IL-13, and IgE) that reflect the host’s immune profile is important for understanding the immune response associated with giardiasis.

The immune response that develops during giardiasis has been reported to be a complex process in which both Th1 and Th2 mechanisms play roles. In experimental models of giardiasis, it has been shown that levels of various cytokines, including IL-4, increase during the course of infection and that the Th2 response makes a significant contribution, particularly to the formation of the mucosal immune response [15]. However, the fact that the parasite can still be eliminated in experimental mouse models lacking IL-4 or IFN-γ demonstrates that neither the Th1 nor the Th2 response alone is protective, and that a balanced T-cell response is necessary for effective parasite control [8,15].

It has been reported that *Giardia* infection induces goblet cell hyperplasia in the intestinal mucosa by increasing antigen-specific Th2 cells, IL-25, and type 2 cytokines, and that this response contributes to the development of chronic carriage via the STAT6 signaling pathway [23]. These findings indicate that *Giardia* can shift the host immune system toward a Th2 response and that IL-4-mediated mechanisms are activated during infection. However, the number of studies evaluating serum IL-4 levels in natural cases of giardiasis in dogs is very limited. In this study, serum IL-4 levels in dogs with giardiasis were significantly higher than those in the healthy control group (Mann–Whitney U = 238.5, Holm-adjusted *p* < 0.001). IL-4 is one of the key Th2 cytokines that promotes the Th2 differentiation of naive CD4+ T lymphocytes, induces class switching in B cells, and plays a role in mucosal defense by increasing goblet cell hyperplasia and mucus production [24]. Therefore, the increase in serum IL-4 observed in our study may indicate that Th2-associated cytokine activity is, at least partly, systemically detectable during canine giardiasis and is associated with *G. duodenalis* infection. Furthermore, this finding appears consistent with the activation of the Th2 response and increases in type 2 cytokines reported during *Giardia* infection [15,23], suggesting that the rise in IL-4 may be an indicator of the immune response accompanying the infection rather than a direct result of parasite elimination [8]. This difference remained statistically significant after Holm correction for multiple comparisons (Hodges–Lehmann median difference 16.31 pg/mL, 95% CI: 9.80–27.47), and the direction and magnitude of the observed difference support IL-4 as a component of the host response to canine giardiasis. These findings should therefore be considered hypothesis-generating and warrant confirmation in larger studies.

IL-13, along with IL-4, is one of the key inflammatory cytokines of the Th2 immune response; it increases mucus secretion by goblet cells, contributes to eosinophil activation, and promotes IgE production in B lymphocytes [24]. However, it has been shown that the IL-13 response may vary depending on the stage of infection, the time of sampling, and its distribution among tissues; in experimental gerbil models, the cytokine profile differs between the early and late stages of infection, and some Th2 cytokines, such as IL-4 and IL-5, increase significantly, particularly in the later stages [18]. Furthermore, in a study on human giardiasis, it was reported that levels of Th2 cytokines, including IL-13, did not differ from those in healthy individuals in children infected with *Giardia* and *Ascaris* [14]. Furthermore, it has been noted that since IL-13 is produced locally in the intestinal mucosa primarily by innate lymphoid cells (ILC2), mast cells, and NKT cells, and exerts its effects largely through autocrine/paracrine mechanisms, serum levels may not always reflect the mucosal immune response [24].

In this study, no statistically significant difference in serum IL-13 levels was observed between dogs with giardiasis and the healthy control group (Mann–Whitney U = 149.5, Holm-adjusted *p* = 0.988). Taken together with the increase in IL-4 levels, this finding suggests that not all components of the immune response are activated simultaneously or to the same degree in the systemic circulation. Furthermore, the results support the hypothesis that serum IL-13 levels may not be a sensitive biomarker for *Giardia* infection and that the biological effects of this cytokine occur primarily locally in the intestinal mucosa. Given the limited statistical power of the present IL-13 comparison, this absence of a significant difference should be interpreted with caution. Nonetheless, it is directionally consistent with the findings reported by Weatherhead et al. [14] in humans, suggesting that changes in IL-13 in the systemic circulation may be limited.

An increase in total IgE levels in parasitic infections is considered one of the most characteristic findings of a Th2 response. It has been reported that IgE production is stimulated because parasite antigens contain allergen-like molecules, and that the resulting IgE plays a role in parasite elimination by binding to FcεRI receptors on mast cells and eosinophils [6,7]. However, findings regarding the serum IgE response in *Giardia* infection are inconsistent. While some studies report elevated total IgE levels in patients with giardiasis [19], other studies have reported that total IgE concentrations in children with giardiasis—particularly those without allergic symptoms—do not differ from those in healthy controls [20,21]. It is suggested that these variations could be caused by factors such as the clinical severity of the infection, host characteristics, and concomitant allergic diseases.

In this study, no statistically significant difference in serum IgE levels was observed between dogs with giardiasis and the healthy control group (Mann–Whitney U = 189.5, Holm-adjusted *p* = 0.125). This finding may be related to the fact that *Giardia*, unlike helminths, is a protozoan that lives non-invasively in the intestinal mucosa, and the immune response that develops during infection is largely confined to the mucosal level [22]. Furthermore, the fact that clinical findings in the dogs in our study were primarily limited to the gastrointestinal tract and that no pronounced allergic symptoms were observed may be another factor explaining the absence of a systemic increase in IgE. Furthermore, since the IgE response is predominantly antigen-specific and localized, it is thought that measuring total serum IgE may not be sufficiently sensitive to reflect the systemic immune response to *Giardia* infection. The results are consistent with the findings of Giacometti et al. [20] and Souza et al. [21], who did not detect significant changes in serum IgE levels in cases of giardiasis without allergic symptoms. However, the very limited number of studies evaluating the relationship between *Giardia* infection and serum IgE levels in dogs suggests that while the current findings may contribute to establishing a species-specific immune response, there is a need for studies with larger sample sizes.

When examined separately by group rather than on the pooled sample (Table 2a,b), IgE, IL-4 and IL-13 were all significantly and positively intercorrelated within the giardiasis group (ρ = 0.64–0.69, *p* < 0.001), whereas in the control group, only IL-4 and IL-13 were significantly correlated (ρ = 0.636, *p* = 0.048); IgE was not significantly correlated with either cytokine in control dogs (ρ ≤ 0.19, *p* ≥ 0.60), although the small control sample (*n* = 10) limits the power to detect such an association. The stronger correlation pattern observed within the giardiasis group is consistent with coordinated variation among these Th2-associated biomarkers; however, the small control sample and exploratory nature of the analysis preclude firm conclusions regarding differences in correlation structure between groups. The strong correlation between IgE and IL-4 observed in the giardiasis group is, nonetheless, consistent with the established role of IL-4 in inducing class switching to IgE in B cells [24], suggesting that this relationship may also exist in dogs during active infection. Our results suggest that evaluating biomarkers individually may not be sufficient. The fact that IL-4 and IL-13 levels change in conjunction with IgE levels within the giardiasis group suggests that these molecules act as components of a Th2 immune response associated with giardiasis, though replication in a larger, independent sample is needed before this pattern can be considered established.

In conclusion, these findings provide partial evidence of altered Th2-associated immune activity in dogs infected with *Giardia duodenalis*, rather than definitively characterizing their systemic Th2 status. However, the significant increase in IL-4 levels, which persisted after correction for multiple comparisons, suggests that canine giardiasis is associated with selective elevation of a Th2-associated cytokine, whereas the absence of significant differences in IL-13 and IgE levels between the infected and control groups indicates that this response may be limited at the systemic level. Similarly, experimental studies have reported that the elimination of *Giardia* requires a balanced and multifaceted cytokine response rather than a response characterized solely by Th1 or Th2 polarization [8,15]. In addition, *Giardia* has been shown to induce antigen-specific Th2 cells and type 2 cytokines in the intestinal mucosa, leading to local immune remodeling [23]. We hypothesize, as an untested explanation, that the effects of IL-13 and IgE may occur predominantly at the intestinal mucosal level; this hypothesis was not directly evaluated in the present study, as no mucosal sampling or local cytokine measurement was performed, and should not be read as a conclusion supported by the present data.

This study has several limitations. Diagnosis was based on a zinc sulfate fecal flotation examination without confirmatory antigen or PCR testing and without assemblage genotyping. Parasite load was not quantified, precluding any dose–response assessment. Commercial ELISA kits were used as supplied by the manufacturer, with the reported coefficients of variation and minimum detectable concentrations taken from the manufacturer’s certificate of analysis; independent validation of species cross-reactivity, standard-curve linearity, or duplicate-well precision was not performed owing to budget constraints. Control dogs were not individually matched to dogs with giardiasis on age, sex, or breed, and no multivariate adjustment for these factors was performed. The comparisons for IgE and IL-13 in particular were statistically underpowered, so the absence of a significant difference cannot be interpreted as evidence of no true difference. Serum samples were collected at a single time point rather than serially across the course of infection, and only serum biomarkers were assessed; the local cytokine response in the intestinal mucosa was not evaluated, and no diagnostic-performance (e.g., ROC) analysis or correlation with clinical severity or outcome was performed.

## Figures and Tables

**Table 1:** Serum Th2-associated biomarker concentrations. Results are expressed as median [interquartile range, IQR].

Variable	Control (*n* = 10), Median [IQR]	Giardiasis (*n* = 30), Median [IQR]	Mann–Whitney U	*p* Value	Holm-Adjusted *p*	Hodges–Lehmann Estimate
IgE (µg/mL)	1.16 [1.02–1.33]	1.32 [1.15–1.62]	189.5	0.062	0.125	0.23 (−0.01 to 0.52)
IL-4 (pg/mL)	23.65 [18.86–27.90]	38.12 [32.55–57.97]	238.5	<0.001	<0.001	16.31 (9.80 to 27.47)
IL-13 (ng/L)	79.05 [65.44–103.68]	78.23 [53.05–103.42]	149.5	0.988	0.988	−0.07 (−27.21 to 21.93)

IgE: immunoglobulin E; IL-4: interleukin 4; IL-13: interleukin 13; CI: 95% confidence interval of the Hodges–Lehmann estimate of the location shift (Giardiasis−Control), calculated from two-sided Mann–Whitney U test (Section 2.5); Mann–Whitney U and *p* values are unadjusted, and Holm-adjusted *p* values control for the three biomarker comparisons.

**Table 2:** (**a**) Correlations among serum Th2-associated biomarker concentrations in dogs with giardiasis (*n* = 30). (**b**) Correlations among serum Th2-associated biomarker concentrations in the healthy control group (*n* = 10).

(**a**)			
	**IgE**	**IL-4**	**IL-13**
IgE	1	0.644 **	0.690 **
IL-4		1	0.672 **
IL-13			1
(**b**)			
	**IgE**	**IL-4**	**IL-13**
IgE	1	0.188	0.030
IL-4		1	0.636 *
IL-13			1

(**a**) IgE: immunoglobulin E; IL-4: interleukin 4; IL-13: interleukin 13. Spearman’s rank-order correlation coefficient (ρ), computed within the giardiasis group only (*n* = 30); ** *p* < 0.01. (**b**) IgE: immunoglobulin E; IL-4: interleukin 4; IL-13: interleukin 13. Spearman’s rank-order correlation coefficient (ρ), computed within the control group only (*n* = 10); * *p* < 0.05.

## Data Availability

The original contributions presented in this study are included in the article. Further inquiries can be directed to the corresponding author.

## Abbreviations

IgE	Immunoglobulin E
IL-4	Interleukin 4
IL-13	Interleukin 13
IQR	Interquartile Range
CI	Confidence Interval
IFN	Interferon
TNF	Tumor Necrosis Factor
STAT6	Signal Transducer and Activator of Transcription 6
ELISA	Enzyme-Linked Immunosorbent Assay
CV	Coefficient of Variation
MDC	Minimum Detectable Concentration
PCR	Polymerase Chain Reaction
ROC	Receiver Operating Characteristic
